# Assessment of mosquito larval productivity among different land use types for targeted malaria vector control in the western Kenya highlands

**DOI:** 10.1186/s13071-015-0968-1

**Published:** 2015-07-05

**Authors:** Eliningaya J. Kweka, Stephen Munga, Yousif Himeidan, Andrew K. Githeko, Guyuin Yan

**Affiliations:** Division of Livestock and Human Health Disease Vector Control, Tropical Pesticides Research Institute, P.O. Box 3024, Arusha, Tanzania; Department of Medical Parasitology and Entomology, School of Medicine, Catholic University of Health and Allied Sciences, P.O. Box 1464, Mwanza, Tanzania; Pan African Mosquito Control Association (PAMCA), P.O. Box 9653, Dar es Salaam, Tanzania; Centre for Global Health Research, Kenya Medical Research Institute, P.O. Box 1578, Kisumu, Kenya; Entomology Unit, Faculty of Agriculture and Natural Resources, University of Kassala, P.O. Box 71, New Halfa, Sudan; Program in Public Health, University of California, Irvine, CA 92697 USA

**Keywords:** Larval habitat, Land use, *Anopheles gambiae* s.s, *Culex quinquefasciatus*, *An. arabiensis*

## Abstract

**Background:**

Mosquito larval source management (LSM) is likely to be more effective when adequate information such as dominant species, seasonal abundance, type of productive habitat, and land use type are available for targeted sites. LSM has been an effective strategy for reducing malaria morbidity in both urban and rural areas in Africa where sufficient proportions of larval habitats can be targeted. In this study, we conducted longitudinal larval source surveillance in the western Kenya highlands, generating data which can be used to establish cost-effective targeted intervention tools.

**Methods:**

One hundred and twenty-four (124) positive larval habitats were monitored weekly and sampled for mosquito larvae over the 85-week period from 28 July 2009 to 3 March 2011. Two villages in the western Kenya highlands, Mbale and Iguhu, were included in the study.

After preliminary sampling, habitats were classified into four types: hoof prints (*n* = 21; 17 % of total), swamps (*n* = 32; 26 %), abandoned goldmines (*n* = 35; 28 %) and drainage ditches (*n* = 36; 29 %). Positive habitats occurred in two land use types: farmland (66) and pasture (58). No positive larval habitats occurred in shrub land or forest.

**Results:**

A total of 46,846 larvae were sampled, of which 44.1 % (20,907) were from abandoned goldmines, 30.9 % (14,469) from drainage ditches, 22.4 % (10,499) from swamps and 2.1 % (971) from hoof prints. In terms of land use types, 57.2 % (26,799) of the sampled larvae were from pasture and 42.8 % (20,047) were from farmland. Of the specimens identified morphologically, 24,583 (52.5 %) were *Anopheles gambiae* s.l., 11,901 (25.4 %) were *Culex quinquefasciatus*, 5628 (12 %) were *An. funestus* s.l. and 4734 (10.1 %) were other anopheline species (*An. coustani, An. squamosus, An. ziemanni* or *An. implexus*). Malaria vector dynamics varied seasonally, with *An.gambiae* s.s. dominating during wet season and *An.arabiensis* during dry season. An increased proportion of *An. arabiensis* was observed compared to previous studies.

**Conclusion:**

These results suggest that long-term monitoring of larval habitats can establish effective surveillance systems and tools. Additionally, the results suggest that larval control is most effective in the dry season due to habitat restriction, with abandoned goldmines, drainage ditches and swamps being the best habitats to target. Both farmland and pasture should be targeted for effective larval control. An increased proportion of *An. arabiensis* in the *An. gambiae* complex was noticed in this study for the very first time in the western Kenya highlands; hence, further control tools should be in place for effective control of *An. arabiensis*.

## Background

*Anopheles gambiae* sibling species have become dominant across Africa, even in areas where these species previously did not exist [1, 2]. New species recently have been identified in the *An. gambiae* complex [3]. Currently, *An. gambiae* s.l. comprises eight species, which have been identified from different geographical locations in sub-Saharan Africa [3, 4]. Previous studies have found *An. gambiae* s.l. increasing in proportion or occurring in areas where it did not previously exist [1, 2].

Land use changes and topography have provided increased exposure to sunlight, which has contributed to the increased availability of potential breeding habitats in the African highlands [5–10]. Reclamation of swamps for agriculture has resulted in more sunlight reaching the swamps, which have then become potential larval habitats [7–9, 11]. Deforestation for agriculture and timber has led to increased availability of new productive *An. gambiae* s.l. breeding sites [12]. Farm and pasture habitats are open and exposed to sunlight, thereby attracting gravid female mosquitoes for oviposition [1, 8, 9, 11, 13, 14]. Increased human population across sub-Saharan Africa has led to increased demand for land, resulting in accelerated land use changes [7, 12]. These land use changes result in microclimatic changes in breeding habitats due to increased temperatures [12, 15–17].

The rise in temperatures within cooler regions of Africa has expanded the distribution of malaria vectors and caused epidemics in highland areas where people are non-immune and have not been exposed to parasite infection [18–20]. In these areas, vector control efforts should be emphasized and implemented in a targeted manner. In the recent past, these areas have received wide coverage of insecticide-treated bed nets (ITNs) together with education on bed net usage [21]. These highland sites have also received wide coverage of indoor residual spray (IRS) [22]. While these strategies may be effective in adult vector control, more emphasis should be placed on controlling the aquatic stages, particularly since the protection offered by current tools in adult vector control has been compromised by intensified insecticide resistance within malaria vector populations [23, 24]. Targeting immature stages and developing monitoring information systems is of paramount importance in effective larval control. In different parts of Africa where larval source management has been practiced, reductions in adult mosquito incidence have been observed [25–29].

The current study monitored larval population dynamics in different land use settings and between different seasons (dry and rainy) for a period of 85 weeks to develop a larval surveillance system in the western Kenya highlands. Additionally, this study assessed the best time of year to conduct efficient larval control in the region.

## Methods

### Study area description

**Fig. 1:**
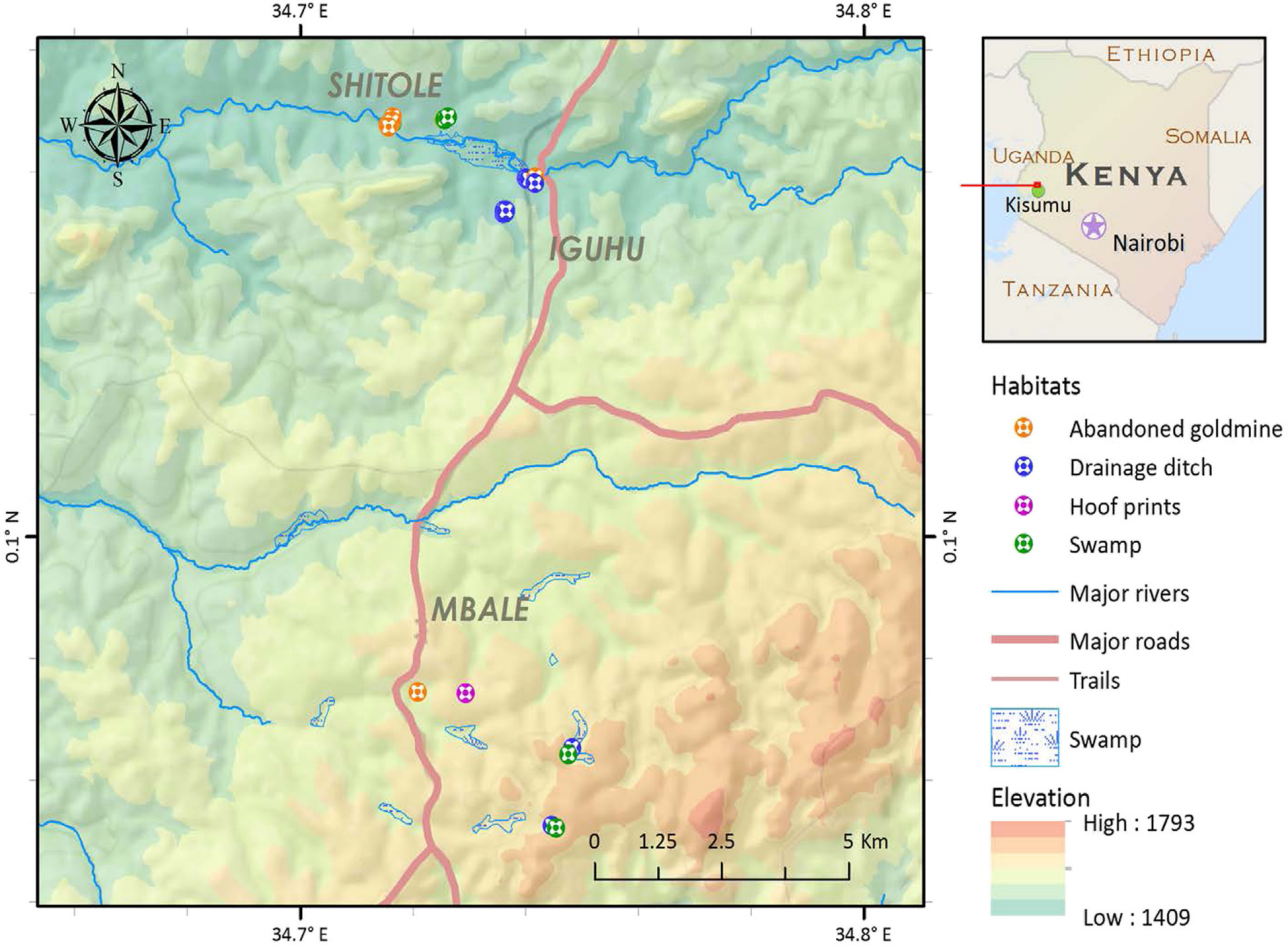
Map of the western Kenya highlands showing habitat types in different topography

This study was conducted in the Mbale and Iguhu constituencies in the western Kenya highlands. Mbale (0^0^. 04′57″N, 34^0^. 43′16″E, 1,620m) and Iguhu (0^0^. 09′41″N, 34^0^. 44′36″E) lie at altitudes of 1,620m and 1,450m above sea level, respectively (Fig. 1). The dry seasons are January to February and temperature ranges between 25 and 30 °C. The main economic activity in these areas is small-scale food and cash crop farming, while a small proportion of inhabitants practice small-scale grazing.

### Definitions of land use and habitat types

Land use types were categorized based on natural vegetation and the activities taking place on the land. The land with the highest tree coverage was considered forest; cultivated land was considered farmland; grazing areas were considered pastureland; and areas of bush land were considered shrub land. All positive habitats (having larvae of either anopheline or culicine species) were categorized as either drainage ditches (canals used to drain water from farms during rainy season), abandoned goldmines (pits left uncovered after mining activities have ceased), swamps (shallow bodies of water with vegetation) or hoof prints (cattle prints on wet ground), according to categorization from previous studies [1, 30]. The majority of these habitats were found in the valley bottom, due to the hilly topography of western Kenya. A total of one hundred and twenty-four (124) habitats were categorized. Positive larval habitats were found in farmland and pasture, while no positive habitats occurred in shrub land or forest. The distribution of habitat types was as follows: drainage ditches (36), hoof prints (21), swamps (32) and abandoned goldmines (35). Hoof prints were the only semi-permanent (short term) larval habitats; all other habitats were permanent. Larval surveys were conducted on all positive habitat types in all land use types.

### Larval surveys, sampling and identification

Preliminary surveys were conducted in all habitats in all land use types using a standard dipper. All positive habitats were considered for follow-up in the main study. Each habitat was geo-referenced using a portable geographical positioning system (GPS). The 124 positive habitats which were selected were sampled for mosquito larvae once a week from 28 July 2009 through 3 March 2011. In habitats with sufficient water volume, a total of twenty (20) dips were made using a standard dipper (350mL, BioQuip Products, Inc. California, USA); for smaller habitats (mostly hoof prints) fewer dips were made. Larval abundance was calculated as the number of larvae per number of dips made in each habitat. Larval surveys were conducted between 10:00 and 13:00 h. All larvae (stages 1 to 4) sampled from each habitat were identified immediately in the field using morphological keys developed by Gillies and Coetzee [31]. Stage 1 and 2 larval instars were returned to their respective habitats while a small number of specimens of stage 3 and 4 larval instars were taken for molecular identification in the laboratory. The larval specimens were preserved in absolute alcohol (70 %) and kept in a freezer at –20 °C until needed for molecular identification. Some larval specimens belonging to *An.gambiae* s.l. and the *An. funestus* group were taken for molecular identification of sibling species by polymerase chain reaction (PCR), following protocols developed by Scott *et al*. for *An.gambiae* s.l. [32] and by Koekemoer *et al*. for *An. funestus* [33].

### Data analysis

Species abundance over time for each habitat type was analysed using generalized linear mixed models (GLMM) with multiple samples collected from each site over time. Larval abundance by land use and habitat type was analysed with GLMM using site as a random effect. Larval habitat and land use types were considered as fixed. Models for each species were developed separately. The abundance of identified *An.gambiae* s.s. and *An. arabiensis* mosquitoes between seasons was compared using one-way analysis of variance (ANOVA) with a significance level of 5 %. Data was analysed using PASW Statistics version 18.0 for Windows (SPSS Inc., Chicago, IL).

### Ethical issues

Ethical approval for this study was granted by the National Ethical Review Committee at the Kenya Medical Research Institute under the main project “Ecology of African highland malaria (II), SSC No. 1382”. Prior to implementation of the study, village leaders and elders were called to a meeting where the essence of the study was explained. Written consent to visit the habitats was obtained from all landowners in the selected sites.

## Results

### Larval abundance and species identification

Among the specimens identified morphologically, 24,583 (52.5 %) were *An. gambiae* s.l., 5628 (12 %) were *An. funestus* s.l., 11,901 (25.4 %) were *Cx. quinquefasciatus* and 4734 (10.1 %) were other anopheline species (*An. implexus, An. squamosus*, *An. ziemanni*, or *An. coustani*). Among the 2350 specimens of *An.gambiae* s.l. identified using molecular techniques, 1445 (61.5 %) were *An.gambiae* s.s., 898 (38.2 %) were *An. arabiensis* and 7 (0.3 %) had no PCR product amplification. Of the 540 specimens in the *An.funestus* group, 172 (31.9 %) were *An. funestus* s.s., 161 (29.8 %) were *An. leesoni*, 68 (12.6 %) were *An. rivulorum* and 137 (25.7 %) were *An. vaneedeni*. Overall larval dynamics of the four main mosquito species (*An.gambiae* s.l., *An. funestus*, *Cx. quinquefasciatus* and other anopheline) showed *An.gambiae* s.l. to dominate over the other species, while *An. funestus* was the least abundant. The dynamics of the identified *An.gambiae* s.s. and *An. arabiensis* displayed similar trends to *An.gambiae* s.l.

In repeated measure analysis using sampling site as a random effect for 85 weeks, larval abundance was found to differ significantly between habitat types (DF = 3, F = 10.117, *P* < 0.0001).

### Habitat and land use types in relation to larval abundance

In the current study, a total of 46,846 larvae were sampled, of which 30.9 % (14,469) were from drainage ditches, 44.6 % (20,907) from abandoned goldmines, 2.1 % (971) from hoof prints and 22.4 % (10,499) from swamps. Eighty-one percent (37,945) were anopheline species and 19 % (8901) were *Cx. quinquefasciatus*. Larval abundance varied by species and habitat type. *An.gambiae* s.l. occurrence showed no significant difference between habitats (DF = 3, F = 0.885, *P* = 0.448). Other anopheline species differed significantly in occurrence between habitats, with the highest occurrence in swamps (DF = 3, F = 14.460, *P* < 0.001). *Cx.quinquefasciatus* occurrence also differed significantly among habitats, again with the highest occurrence in swamps (DF = 3, F = 5.214, *P* = 0.001). *An. funestus* occurrence differed significantly between habitat types, with the highest abundance occurring in drainage ditches (DF = 3, F = 9.765, *P* < 0.001). No *An. funestus* larvae were recorded from hoof prints.

When analysed by land use type, 42.8 % (20,047) of the sampled specimens were from farmland and 57.2 % (26,799) from pasture. *An. gambiae* s.l. was statistically more abundant in pasture than in farmland (DF = 1, F = 4.824, *P* = 0.028), as was *An. funestus* (DF = 1, F = 5.133, *P* = 0.024). Other anopheline species had no significant difference in occurrence between pasture and farmland (DF = 1, F = 0.001, *P* = 0.979); nor did *Cx. quinquefasciatus* (DF = 1, F = 4.824, *P* = 0.143).

### Anopheline larval abundance by seasonality

**Fig. 2:**
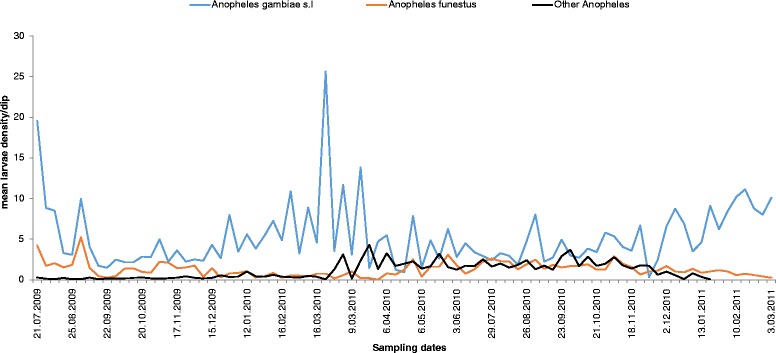
Dynamics and occurrence peaks of immature stages of *Anopheles gambiae* s.l., *An. funestus* and other anopheline species in the western Kenya highlands over a period of 85 weeks

Among anopheline species sampled, there were significant differences in larval abundance by season, with *An.gambiae s.l* larvae more abundant in dry season than in rainy season (F = 17.76, df = 1, *P* ≥ 0.001) and *An.funestus* larvae more abundant in rainy season than in dry season (F = 4.16, df =1, *P* = 0.045). Other anopheline larvae were also significantly more abundant in rainy season than in dry season (F = 5.64, DF = 1, *P* = 0.020) (Fig. 2). Larval density varied significantly among habitats between weeks (DF = 3, F = 27.18, *P* < 0.0001).

## Discussion

This study has shown that extending larval habitat follow-up for longer periods of time can provide critical information for larval source management. Such information includes habitat type, larval abundance, species composition and dynamics, seasonality, and land use type. It is interesting to note that, compared to historical findings, this study found a higher proportion of *An. arabiensis* abundance (38.2 %) in the study area. In a previous study by Wamae *et al*. at the same sites, the proportion of *An.arabiensis* was reported to be 2.6 % among all *An.gambiae* s.l. sampled [34]. It is possible that changes in land cover and topography in the western Kenya highlands have increased the suitability of breeding sites for *An.arabiensis*, as was found in previous studies [7, 12, 35]. A higher proportion of *An.arabiensis* (38.2 %) has been found in larval habitat compared to that found in indoor sampling using CDC light traps and pyrethrum spray catches (2.6 %) [36]. Our current results show that we can assess mosquito species composition with greater accuracy by establishing larval habitat monitoring surveillance systems. This is important, as sampling methods such as CDC light traps, pyrethrum spray catches and window/eave traps have been compromised by the wide coverage of ITN and IRS programmes [37].

In this study, *Anopheles* and *Culex* species were found in all four habitat types (hoof prints, abandoned goldmines, swamps and drainage ditches) with the exception of *An. funestus*, which was not found in hoof prints. Naturally, *An. funestus* breeds in large water bodies which are permanent with shade and vegetation [38, 39]. Other studies conducted at the same sites have shown similar species composition across habitat types, with *An. funestus* also not recorded from hoof prints [6–8, 11, 13, 40]. In designing an effective control tool for malaria vectors, the major target habitats should be abandoned goldmines, swamps and drainage ditches which are open to sunlight. These habitats attract more *An. gambiae* s.l. mosquitoes to oviposit, leading to a higher abundance of larvae than in other habitat types. Also, *An.gambiae* s.l. was observed in all habitats except swamps during the dry season, a result of restricted water sources. *Cx.quinquefasciatus* colonized swamps, drainage ditches and abandoned goldmines during dry seasons when these habitats had high rates of organic matter and plant decomposition; by contrast, such characteristics have been found to cause *An.gambiae* s.l. to avoid breeding in matured habitats [41]. However, *Cx. quinquefasciatus* has been found to be only a nuisance vector in the study area, not a vector for disease parasites. *An. gambiae* s.l. has been found to colonize fresh shallow and temporary habitats with relatively low grass cover and high sunlight exposure [1, 11]. This information about species composition and habitat preference is of paramount importance in developing cost-effective and efficient larval control. In Brazil, Killeen and others showed that a well-designed larval control programme can contribute to the elimination of *An.gambiae* [42]. Long-term monitoring of larval habitats has been found to have a positive impact on our understanding of habitat productivity and larval abundance [1, 11].

Malaria vector and non-vector species were found in both of the land use types that had positive breeding sites (i.e., farmland and pasture). Comparison of larval abundance between the two land use types shows a higher abundance in pasture than in farmland. Previous studies have found higher larval abundance in farmland than in pasture [9, 11]. The differences in larval abundance between the two land use types might have contributed to the increased proportion of *An.arabiensis*, which can take blood meals from grazing cattle, rest in bushes and, soon after digestion, oviposit in nearby habitats. This maybe a survival strategy and adaptation behaviour for *An.arabiensis* in this highland site in western Kenya. The coverage of pyrethroids used in IRS and ITNs in western Kenya may also shift species composition in favour of *An.arabiensis*, since the availability of an alternative blood meal allows *An. arabiensis* mosquitoes to avoid exposure to the current intervention tools. In other parts of Kenya, similar scenarios have been found. After massive coverage with intervention tools there was a tremendous shift in malaria vector species composition from *An.gambiae* s.s. to *An.arabiensis* [43]. The same trend was observed in Tanzania in areas of intensive bed net coverage [44]. Since *An.gambiae* s.s. mosquitoes feed indoors (mostly on humans) and rest indoors where intervention coverage exists, their blood meal seeking time is increased and survivorship is reduced [45]; hence, the population of zoophilic and exophilic *An.arabiensis* overlaps and dominates. This suggests the possibility of outdoor malaria transmission in this area of western Kenya where the population of *An.arabiensis* is escalating. The only option for reducing outdoor vector populations is to target larval source management, which is possible when efficient and effective surveillance systems are established. It is plausible that outdoor malaria vector populations have increased as a result of induced exophily due to IRS and ITN coverage [46, 47].

In larval habitat monitoring and control, understanding seasonal abundance and species composition is of great importance when designing efficient, cost-effective tools. Seasonality has always been associated with the abundance of immature stages and adult mosquitoes [48]. The abundance of both larvae and adult malaria vectors has been observed to increase soon after the rainy season with the stabilization of habitats [49]. In this study, seasonality has been demonstrated to influence larval abundance for all *Anopheles* species found in the study site, with the dry season having statistically significantly higher larval abundance than the rainy season. This can be attributed to the flush effect during the rainy season, which may result in unexpected larval loss in habitats [1, 11, 49]. However, the population can proliferate over time, reaching its peak during the beginning of dry season when weather is favourable and the shortened gonotrophic cycle leads to high larval density. This in turn is likely to increase the population of adults, which are then able to lay higher densities of eggs when habitats are completely well established. The topography of western Kenya is undulating, and larval habitats occur mostly in the valley bottoms, which are easily flooded during the rainy season [1, 49]. The dry season has been shown to have more larval abundance for all species. This can be attributed to stable habitats, which favour oviposition by gravid females and provide enough food for larvae survivorship [14, 50]. During dry season, habitats are few and exposed to sunlight, hence facilitating microbial decomposition activity and photosynthetic algal growth, which in turn provides food to mosquito larvae [1, 11, 30, 51]. Additionally, during dry season habitats maintain stable temperature, which has been found to shorten larval immature stages [52]. In this study larval habitats in dry season were few, but each site contained high densities of larvae of all species found in that site. Thus this long-term study has shown that dry season is the best time to target for effective larval habitat control. Habitats are restricted and few, so follow-up management will be effective with regard to time, space, cost and expected outputs. In previous studies, the best time for larval control in this study area was suggested to be between the short rainy season and the dry season [1, 11]. The current study suggests the same but with more detailed habitats, land use patterns and larval abundance dynamics. If control programmes are coordinated in these areas during dry season, larvicidal efficacy will be higher, as there is no larvicidal dilution effect and larval density is at its peak. Monitoring is likely to be easier, as habitats are few and restricted. From different studies conducted across Africa, it is evident that if larval source management is targeted properly, malaria control and elimination can be achieved [53]. Community willingness to be involved in malaria control has also shown a positive impact and should continue to be cultivated and extended throughout sub-Saharan Africa [54–56].

## Conclusion

These results suggest that long-term monitoring of larval habitats can establish effective surveillance systems and tools. Additionally, the results suggest that larval control is most effective in the dry season due to habitat restriction, with abandoned goldmines, drainage ditches and swamps being the best habitats to target. The land use type that should be targeted are both pasture and farmlands. An increased proportion of *An. arabiensis* in the *An. gambiae* complex was noticed in this study for the very first time in the western Kenya highlands; hence, further control tools should be in place for effective control of *An. arabiensis*.
